# The Receptor Tyrosine Kinase Axl in (Advanced) Gastric Cancer—From Pathophysiology to Therapeutic Impact

**DOI:** 10.3390/medicina61091619

**Published:** 2025-09-08

**Authors:** Oliver Daniel Schreiner, Thomas Gabriel Schreiner, Lucian Miron, Romeo Cristian Ciobanu

**Affiliations:** 1 Department of Medical Specialties III, Faculty of Medicine, “Grigore T. Popa” University of Medicine and Pharmacy, 700115 Iasi, Romania; schreiner.oliver-daniel@d.umfiasi.ro (O.D.S.); lucian.miron@umfiasi.ro (L.M.); 2 Medical Oncology Department, Regional Institute of Oncology, 700483 Iasi, Romania; 3 Department of Electrical Measurements and Materials, Gheorghe Asachi Technical University, 700050 Iasi, Romania; 4 First Neurology Clinic, “N. Oblu” Clinical Emergency Hospital, 700309 Iasi, Romania

**Keywords:** gastric cancer, receptor tyrosine kinase Axl, molecular mechanisms of Axl in tumors, cancer treatment targeting Axl

## Abstract

*Background*: Gastric cancer (GC) is a leading cause of cancer-related mortality worldwide, with its advanced stages presenting significant challenges for the clinical oncologist. Axl is a member of the TAM family of receptor tyrosine kinases that is becoming increasingly important in the pathophysiology of (advanced) GC. This receptor, activated by its ligand Gas6 (growth arrest-specific gene 6), is implicated in various oncogenic processes, including cell survival, proliferation, migration, and immune evasion. Overexpression or aberrant activation of Axl has been associated with poor prognosis, tumor aggressiveness, and resistance to conventional therapies in gastric cancer. *Objectives*: This review aims to consolidate current knowledge on Axl’s role in gastric cancer pathophysiology and explore its therapeutic implications. *Materials and Methods*: A thorough search was conducted in the most relevant online databases, using different combinations of the following terms: Axl, GC, pathophysiology, and therapeutic target. *Results*: In the first part, the molecular mechanisms of Axl in tumors, which involve, among others, the activation of downstream signaling pathways, including PI3K/AKT, MAPK/ERK, and NF-κB, are discussed. Subsequently, potential treatments targeting Axl and potential combination therapies are highlighted, based on the encouraging results from preclinical and clinical studies. Finally, as the Axl–tumor microenvironment interplay is discussed, with therapeutic implications, it thus opens new pathways for research on effective treatments in advanced gastric cancer. *Conclusions*: Understanding Axl’s role in the pathophysiology of GC is essential to develop efficient targeted therapies with improved clinical effects.

## 1. Introduction

Gastric cancer (GC) remains a major global health concern, ranking as the fifth most common malignancy and the fourth leading cause of cancer-related mortality worldwide [1]. Despite a decline in incidence in certain regions, particularly in Western countries, GC continues to pose a significant burden in East Asia, Eastern Europe, and parts of South America [2]. The prognosis for patients with advanced or metastatic disease remains dismal, with five-year survival rates rarely exceeding 10–20% according to mortality data collected by the National Center for Health Statistics in the United States population in 2020, largely due to late diagnosis, inherent tumor heterogeneity, and limited effectiveness of currently available therapies [3]. Current molecular stratification in gastric adenocarcinoma follows the TCGA framework, including: EBV-positive tumors (~9%) which show striking immune activation with frequent PIK3CA/ARID1A mutations and 9p24.1 (PD-L1/PD-L2) amplification [4], MSI-high/dMMR tumors (~20% in Western cohorts) are hypermutated, carry abundant neoantigens, are associated with a favorable prognosis, and have tumor-agnostic approvals for PD-1 inhibitors [5], genomically stable (GS) cancers are enriched for diffuse/Lauren type with CDH1 and RHOA alterations and recurrent CLDN18–ARHGAP fusions [6], and chromosomal instability (CIN) tumors display aneuploidy and receptor tyrosine kinase (RTK) amplifications [6]. In parallel, HER2-positive disease remains the canonical, clinically actionable segment: first-line therapy consists of trastuzumab plus chemotherapy, and for patients with PD-L1 CPS ≥ 1, adding pembrolizumab improves overall survival (KEYNOTE-811), which is now incorporated into approvals/guidelines [7]. In this context, there is an urgent need for improved molecular characterization of GC to guide the development of novel, targeted therapeutic strategies.

RTKs play crucial roles in regulating cell proliferation, survival, migration, and differentiation [8]. Aberrant RTK signaling—whether through overexpression, mutation, or autocrine activation—has been implicated in the pathogenesis and progression of various cancers, including gastric cancer [9]. Several RTKs, such as Human Epidermal Growth Factor Receptor 2 (HER2), Mesenchymal–Epithelial Transition Factor (MET), and Fibroblast Growth Factor Receptor 2 (FGFR2), have already emerged as potential therapeutic targets in GC, underscoring the clinical relevance of this receptor class [10]. Nevertheless, therapeutic resistance and limited patient stratification highlight the need to investigate additional RTKs that may contribute to disease progression and represent viable targets for intervention.

Axl, a member of the TAM family of RTKs, which additionally comprises Tyro3 and MerTK, has garnered increasing attention for its multifaceted roles in oncogenesis [11]. Initially identified in myeloid leukemia, Axl is now recognized as a key mediator of epithelial-to-mesenchymal transition (EMT), immune evasion, therapeutic resistance, and metastasis across a broad spectrum of malignancies [12,13]. Its activation, primarily through binding of the ligand growth arrest-specific 6 (Gas6), triggers downstream signaling pathways including PI3K/AKT, MAPK, and NF-κB, which collectively contribute to tumor aggressiveness and poor clinical outcomes [14]. Although the role of Axl has been explored in several cancers, its pathophysiological and therapeutic relevance in gastric cancer remains underappreciated.

This review aims to provide a comprehensive overview of Axl signaling in gastric cancer, with a focus on its contribution to tumor progression, immune modulation, and drug resistance. The current understanding of Axl expression and function in GC is summarized, highlighting its potential as a prognostic biomarker and evaluating emerging therapeutic strategies targeting Axl, particularly in the context of advanced disease. By elucidating the multifaceted role of Axl in gastric cancer biology, this review aims to inform future research directions and promote the development of more effective, targeted treatment approaches.

## 2. Materials and Methods

### 2.1. Literature Search Strategy

A comprehensive literature search was performed in PubMed/MEDLINE, Web of Science, and Embase, covering publications from January 2000 to May 2025. The search strategy combined controlled vocabulary and free-text terms related to “gastric cancer,” “stomach neoplasms”, “Axl”, “receptor tyrosine kinase”, “immune modulation”, “drug resistance”, and “targeted therapy”. Additional references were identified by manually screening the bibliographies of relevant reviews and included articles. The review was conducted in accordance with the Preferred Reporting Items for Systematic Reviews and Meta-Analyses extension for Scoping Reviews (PRISMA-ScR).

### 2.2. Eligibility Criteria (PICOTS Framework)

- Population (P): Patients with gastric or gastroesophageal junction adenocarcinoma.
- Intervention (I): Studies evaluating Axl expression, signaling pathways, or therapeutic targeting of Axl (e.g., small-molecule inhibitors, monoclonal antibodies, antibody–drug conjugates).
- Comparator (C): Non-tumor gastric tissue, Axl-negative cohorts, standard of care, or alternative targeted therapies (when applicable).
- Outcomes (O): Biological function of Axl in tumor progression, immune modulation, and drug resistance; prognostic or predictive biomarker value; efficacy and safety outcomes of Axl-targeting strategies.
- Timing (T): No restriction on follow-up duration.
- Setting (S): Preclinical (cell line, animal models) and clinical studies (retrospective/prospective cohorts, trials).

### 2.3. Study Selection

All articles retrieved from the initial search were checked for duplication, with titles and abstracts being screened independently by two reviewers. Full-text articles were then assessed for eligibility. The inclusion criteria were represented by original research articles (preclinical or clinical), clinical trials, and translational studies reporting on Axl in gastric cancer. Information from relevant new reviews was also included, particularly in the section discussing Axl’s role in gastric cancer. Exclusion criteria consisted of conference abstracts without full text, non-English articles, studies on other tumor entities without data specific to GC, and studies with insufficient methodological detail.

### 2.4. Data Extraction and Reviewer Agreement

A standardized extraction sheet was used to collect data on study characteristics (authors, year, design, population, methods, key findings). Two reviewers (O.D.S. and T.G.S.) independently extracted data. Discrepancies were resolved by discussion; if consensus was not reached, a third reviewer (L.M.) adjudicated.

### 2.5. Limitations of the Scoping Review

The present review has several limitations that warrant consideration. First, the available body of evidence on Axl signaling in gastric cancer is limited, with many mechanistic insights and therapeutic findings extrapolated from other malignancies. While these cross-tumor comparisons provide valuable hypotheses, they cannot substitute for gastric cancer–specific validation. Second, most data in gastric cancer derive from preclinical models or small patient cohorts, with a lack of large-scale genomic, transcriptomic, and clinical correlation studies. Third, the therapeutic relevance of Axl blockade in gastric cancer remains speculative, as clinical trial data are still largely absent. Finally, as a scoping review, our work was designed to summarize the breadth of evidence rather than systematically assess the quality of studies or the risk of bias. These limitations highlight the urgent need for dedicated mechanistic and translational studies in gastric cancer to establish whether Axl truly represents a viable biomarker and therapeutic target in this disease.

## 3. Axl’s Role in Gastric Cancer

### 3.1. Axl Structure and Ligands

**Figure 1:**
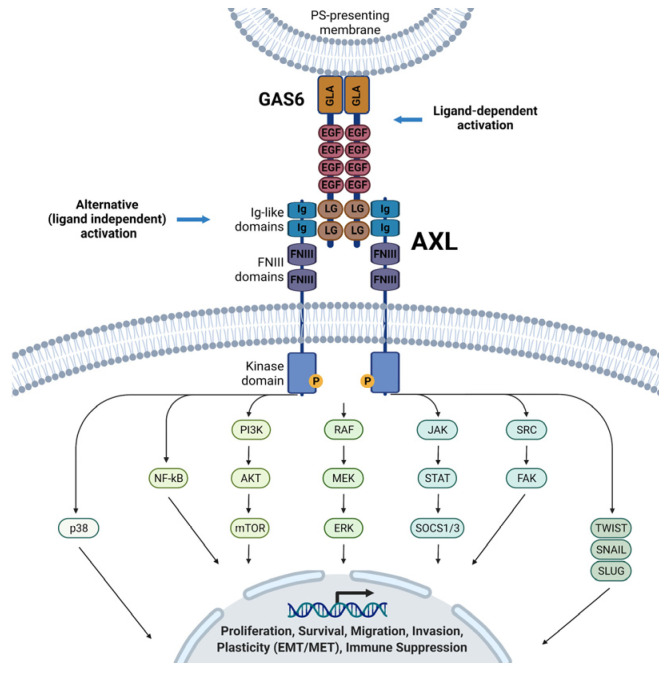
Axl structure and functional pathways in gastric cancer (used with agreement from Engelsen et al. [12]).

Axl is a member of the TAM family of receptor tyrosine kinases, characterized by their conserved domain architecture and shared ligands. The Axl receptor consists of an extracellular domain, a single transmembrane region, and an intracellular tyrosine kinase domain [15]. The extracellular region comprises two immunoglobulin (Ig)-like domains and two fibronectin type III (FNIII) domains, which facilitate ligand binding and receptor dimerization [16]. Figure 1 illustrates the structure of the Axl receptor.

The primary and best-characterized ligand for Axl is growth arrest-specific 6 (Gas6), a vitamin K-dependent protein that is structurally related to protein S [17]. Gas6 binds with the highest affinity to Axl among the TAM family members and significantly lowers affinities for Tyro3 and MerTK [18]. The interaction between Gas6 and Axl is calcium-dependent, leading to receptor dimerization and autophosphorylation of tyrosine residues within the intracellular kinase domain [18]. This, in turn, activates multiple downstream signaling pathways, including phosphatidylinositol 3-kinase (PI3K)/AKT, mitogen-activated protein kinase (MAPK), NF-κB, and signal transducer and activator of transcription (STAT) pathways [19].

In addition to Gas6, recent studies have identified alternative ligands and co-regulators of Axl, including protein S and apoptotic cell-derived phosphatidylserine complexes [20]. However, these are less potent in activating Axl signaling. Soluble Axl (sAxl), generated through proteolytic cleavage of the extracellular domain, acts as a decoy receptor and may modulate Gas6 bioavailability in the tumor microenvironment, thereby influencing Axl activity [21].

Understanding Axl’s structural features and ligand-binding dynamics is critical for the rational design of targeted therapies. Several therapeutic agents, including small-molecule inhibitors and monoclonal antibodies, aim to disrupt Axl signaling by either preventing ligand-receptor interaction or inhibiting receptor autophosphorylation, highlighting the clinical relevance of this pathway in oncogenesis.

### 3.2. Axl in Cancer Pathophysiology

Axl plays a multifaceted role in the pathophysiology of cancer, including GC, where it contributes to several processes in the onset and evolution of the malignancy. Its activation drives key biological processes that support tumor progression, therapeutic resistance, and immune evasion.

Firstly, Axl activation supports cancer cell proliferation and survival primarily through the PI3K/AKT and MAPK/ERK pathways, which regulate cell cycle progression and resistance to apoptosis [22]. The activation of Axl triggers the recruitment of the p85 regulatory subunit of PI3K, leading to the activation of PI3K, which in turn phosphorylates and activates AKT, a key kinase in the PI3K/AKT pathway [23]. Regarding the MAPK/ERK pathway, Axl activation facilitates the recruitment of Grb2, an adaptor molecule that links Axl to the MAPK/ERK pathway, which ultimately leads to the activation of ERK, a downstream effector of the MAPK pathway [24].

In GC cell lines, Axl overexpression is correlated with an enhanced proliferative capacity and resistance to cytotoxic stress [25]. Mechanistically, Axl signaling promotes cyclin D1 expression and inhibits pro-apoptotic factors such as BAX, tipping the balance toward cell survival [26]. Moreover, Axl can confer resistance to apoptosis induced by chemotherapeutic agents, including cisplatin and 5-fluorouracil, by maintaining activation of AKT and STAT3 [27]. This anti-apoptotic effect is particularly relevant in the context of advanced GC, where treatment options are often limited by drug resistance.

Regarding migration and invasion, Axl is a key driver of epithelial-to-mesenchymal transition (EMT), a process crucial for tumor invasion and metastasis [28]. Through EMT, epithelial cancer cells acquire mesenchymal traits that enhance motility and invasiveness. Axl activation induces EMT markers such as vimentin, N-cadherin, and Snail, while downregulating E-cadherin, thereby promoting a migratory phenotype [29].

In gastric cancer models, Axl knockdown or inhibition has been shown to suppress cell migration and invasion in vitro, accompanied by a reversal of EMT-associated transcriptional profiles [16]. These effects are mediated in part through activation of small GTPases (e.g., Rac1, RhoA) and focal adhesion kinase (FAK), which coordinate cytoskeletal remodeling and cell movement [30].

Axl signaling also contributes to tumor angiogenesis, a vital process for sustained tumor growth and dissemination. Axl enhances the expression of pro-angiogenic factors such as vascular endothelial growth factor (VEGF) and interleukin-8 (IL-8), facilitating endothelial cell proliferation and new vessel formation [31]. Crosstalk between tumor cells and stromal components, including endothelial cells and pericytes, is partly mediated by Axl, with Axl a central player in the angiogenesis of gastric tumors [32].

In vivo studies using xenograft models of GC have demonstrated that Axl inhibition leads to reduced microvessel density and impaired tumor vascularization, further supporting its role in angiogenesis [33].

Finally, one of the most clinically relevant functions of Axl is its ability to drive therapeutic resistance. Axl expression is frequently upregulated in response to cytotoxic chemotherapy, targeted agents, and even immune checkpoint blockade, suggesting a role in adaptive resistance mechanisms [34]. In GC, Axl overexpression has been linked to resistance against trastuzumab in HER2-positive tumors, as well as to acquired resistance to tyrosine kinase inhibitors (TKIs) targeting MET or FGFR2 [35]. Axl can heterodimerize with HER2, leading to the activation of downstream pathways, including PI3K/AKT and MAPK, thereby promoting cell survival and proliferation and ultimately counteracting the effects of trastuzumab [35]. This resistance can occur both inherently and through the development of acquired resistance.

**Figure 2:**
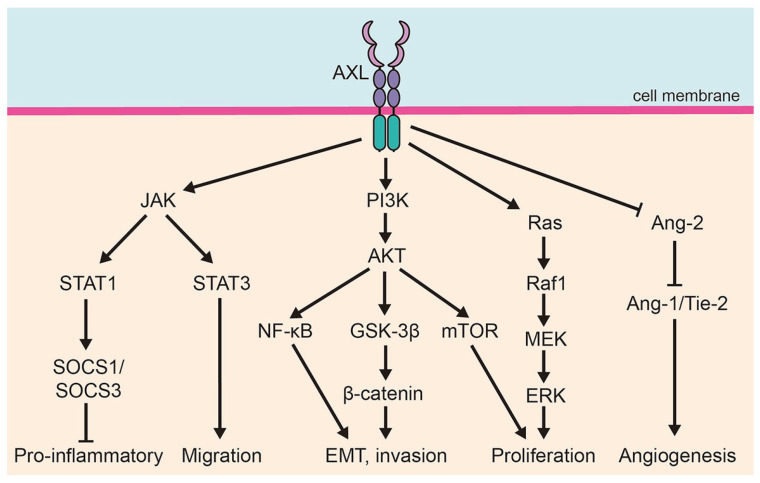
Axl-related pathways and the most relevant biological processes, applicable also in the case of gastric cancer (used with agreement from Tang et al. [15]).

Mechanistically, Axl activation sustains pro-survival signaling and may induce a stem-like phenotype characterized by increased aldehyde dehydrogenase (ALDH) activity, sphere-forming capacity, and expression of stemness-associated transcription factors such as Sox2 and Nanog. These features contribute to tumor heterogeneity and plasticity, hallmarks of aggressive and treatment-refractory gastric cancers. Table 1 summarizes the most relevant roles of Axl in cancer pathophysiology, while Figure 2 graphically illustrates the most relevant Axl-related pathways.

### 3.3. Correlation with Clinical Outcomes

The clinical significance of Axl expression in GC has been increasingly recognized, particularly in the context of disease aggressiveness, therapeutic resistance, and overall patient outcomes. Accumulating evidence demonstrates a consistent association between elevated Axl expression and adverse clinical features in GC. Immunohistochemical analyses of resected GC specimens have shown that Axl is overexpressed in approximately 30–50% of cases, though prevalence rates vary depending on the detection method and cohort composition [36]. High Axl expression is significantly associated with several aggressive pathological features, including deeper invasion depth (T3–T4), lymphovascular invasion, perineural infiltration, lymph node metastasis, and advanced TNM stage (III–IV) [37]. Additionally, Axl-positive tumors are more likely to exhibit poorly differentiated or diffuse-type histology, characteristics known to portend a worse prognosis. In several studies, tumors with high Axl expression also showed greater microvessel density and enhanced expression of mesenchymal markers, consistent with an EMT phenotype [38]. These findings suggest that Axl may serve not only as a molecular marker of invasiveness and metastatic potential but also as a surrogate for histological and molecular aggressiveness.

Numerous studies have evaluated the prognostic impact of Axl expression in GC, one topic being the strong association between Axl overexpression and adverse prognostic features in GC. Notably, He et al. demonstrated that Axl and its ligand Gas6 are significantly upregulated in GC cell lines compared to normal gastric epithelial cells. Using Kaplan–Meier survival analysis, they showed that high Axl expression was significantly associated with reduced overall survival in GC patients (HR ≈ 2.2; *p* < 0.001), supporting its role as a negative prognostic marker [38].

Building on these observations, a more recent study by Zhou et al. analyzed transcriptomic data from The Cancer Genome Atlas (TCGA) and confirmed that elevated AXL mRNA levels correlated with poor clinical outcomes in a cohort of 375 patients with gastric adenocarcinoma. Multivariate Cox regression analysis identified high AXL expression as an independent predictor of overall survival, irrespective of clinicopathological variables [39]. Immunohistochemical validation in a separate tissue microarray cohort further supported the relevance of Axl at the protein level. Additionally, a comprehensive meta-analysis by Wu et al. reinforced these findings by evaluating over 3000 patients across 25 studies, including gastric cancer. Axl overexpression was consistently associated with worse overall (HR ≈ 2.03) and disease-free survival (HR ≈ 1.85), as well as advanced TNM staging, lymph node involvement, and distant metastasis [40]. Although this analysis encompassed multiple solid tumors, the consistency of the prognostic impact across cancers underscores the clinical significance of Axl expression. Taken together, these studies support the role of Axl not only as a biomarker of aggressive tumor biology but also as a potential tool for risk stratification and prognostication in advanced GC.

A final interesting and practically oriented aspect is related to the soluble form of Axl (sAxl), generated by proteolytic cleavage of the extracellular domain, which has emerged as a promising non-invasive biomarker. SAxl can be detected in peripheral blood, providing a dynamic readout of Axl pathway activity in real-time. Although its clinical utility in gastric cancer remains largely unexplored, mounting evidence from other solid tumors supports its potential relevance in GC. In hepatocellular carcinoma (HCC), elevated serum sAxl levels have been associated with tumor burden, vascular invasion, and poor prognosis. Reichl et al. demonstrated that sAxl levels were significantly higher in HCC patients compared to cirrhotic and healthy controls, and levels increased in tandem with disease progression and metastatic spread. Importantly, high pre-treatment sAxl predicted reduced overall survival, and dynamic changes in sAxl were reflective of treatment response, suggesting its potential for longitudinal disease monitoring [40]. Similar findings have been reported in melanoma. A study by Flem-Karlsen et al. showed that sAxl concentrations were significantly higher in patients with advanced-stage melanoma, correlating with tumor Axl expression and poor therapeutic outcomes, particularly in those treated with immune checkpoint inhibitors [41]. Moreover, in pancreatic ductal adenocarcinoma (PDAC), Martinez-Bosch et al. found that sAxl distinguished malignant from benign pancreatic disease more accurately than CA 19-9, the standard marker, and predicted survival outcomes [42]. Although no large-scale studies have yet validated sAxl in GC, its established role in other aggressive malignancies—many of which share Axl-driven mechanisms of progression, invasion, and immune modulation—suggests it could serve as a surrogate marker for tumor burden and therapeutic response in GC as well. Several factors may explain the discrepancy between the consistent association between Axl overexpression and aggressive clinicopathological features in GC and the lag in translation into clinical trials. First, the clinical development of Axl-targeted agents has been largely prioritized in cancers with a higher incidence or well-established preclinical models, such as non-small-cell lung cancer, breast cancer, and pancreatic cancer. Furthermore, the molecular heterogeneity of GC may complicate patient selection and hinder the identification of clear signals of efficacy without biomarker-driven stratification. Lastly, the relative scarcity of prospective clinical datasets in GC has limited the ability to validate Axl as a therapeutic biomarker or to justify large-scale trial investments.

Still, Axl’s ability to be repeatedly measured from serum makes it an attractive candidate for real-time monitoring, particularly in advanced disease, where tissue access is often limited. Prospective studies in GC are needed to define the kinetics, specificity, and prognostic value of sAxl. Nevertheless, early data provide a compelling rationale for investigating sAxl as a circulating biomarker in the context of gastric cancer. Table 2 summarizes the most relevant correlations between Axl status and the clinical outcome.

**Table 2:** Correlations between Axl status and clinical outcome in gastric cancer (↑—increase in value).

Axl Status/Biomarker	Main Findings/Correlations	Clinical Implications	Relevant References
High Axl expression (IHC, ~30–50% of GC cases)	Associated with advanced pathological features: deeper invasion (T3–T4), lymphovascular and perineural invasion, lymph node metastasis, higher TNM stage (III–IV), poorly differentiated/diffuse histology, EMT phenotype (↑ mesenchymal markers, ↑ microvessel density).	Marker of aggressiveness, invasiveness, and metastatic potential.	[36,37,38]
High Axl expression (tumor tissue—survival data)	Kaplan–Meier analysis: significantly reduced overall survival (HR ~2.2, *p* < 0.001). Independent negative prognostic marker by multivariate Cox regression in TCGA cohort.	Prognostic biomarker for poor outcome.	[38,39]
Meta-analysis (25 studies, >3000 patients, including GC)	Axl overexpression correlated with worse OS (HR ≈ 2.03) and DFS (HR ≈ 1.85), and advanced TNM stage, LN involvement, distant metastasis.	Broadly supports Axl as a pan-cancer poor prognostic factor, validated in GC.	[40]
Soluble Axl (sAxl, serum)	Elevated levels linked to tumor burden, vascular invasion, poor survival, and treatment response in HCC, melanoma, PDAC. Not yet validated in GC, but mechanistically plausible.	Promising non-invasive biomarker for real-time disease monitoring in GC (prospective validation needed).	[41,42,43]

## 4. Therapeutic Targeting of Axl in Gastric Cancer

### 4.1. Axl Inhibitors in Development

The blockade of Axl signaling represents a promising therapeutic avenue in GC, particularly in combating chemoresistance, metastasis development, and immune evasion. Pharmacological strategies include small molecules targeting the kinase domain of Axl, monoclonal antibodies directed against Axl or its ligand Gas6, and combination treatments integrating Axl inhibition with chemotherapy, immunotherapy, or other targeted agents.

Among the small molecule inhibitors, worth mentioning are Bemcentinib (R428/BGB324) and TP-0903. R428 is the most extensively studied selective Axl inhibitor, with an IC_50_ of ~14 nM, demonstrating greater than 50-fold selectivity over other kinases [44]. Preclinical models in breast cancer have shown that oral R428 reduces metastasis, represses EMT markers, and synergizes with cisplatin to curb micrometastases and prolong survival [45]. Intriguingly, independent of Axl activity, R428 induces lysosomal dysfunction and autophagy-mediated apoptosis, suggesting broader anti-cancer effects [46]. Although phase II trials are ongoing in solid tumors, including non-small cell lung cancer (NSCLC) [44], triple-negative breast cancer (TNBC) [47], acute myeloid leukemia (AML) [48], and melanoma, data in GC are limited. However, the mechanistic similarity and action across multiple tumor contexts support exploration in GC. TP-0903 is an orally available Axl inhibitor with activity against Aurora B and JAK2 [49]. In pancreatic cancer models, TP-0903 alone modestly reduced tumor burden; however, when combined with chemotherapy and anti–PD-1, the triple regimen significantly limited primary growth, reprogrammed tumors toward an epithelial phenotype, reduced macrophage-mediated immunosuppression, and prolonged survival [49]. Importantly, in human peripheral blood mononuclear cells (PBMCs) from early-phase trial participants, TP-0903 reduced the Treg population and induced Th1 polarization, foreshadowing immunomodulatory benefits in GC [50]. These findings highlight TP-0903’s dual role in reversing EMT and enhancing antitumor immunity, both relevant to advanced GC biology. Other Axl kinase inhibitors, such as LDC1267 and NPS-1034 [51], targeting multiple TAM family members, have shown preclinical efficacy in models of metastasis and treatment resistance, though their utility in GC remains explicitly untested.

**Figure 3:**
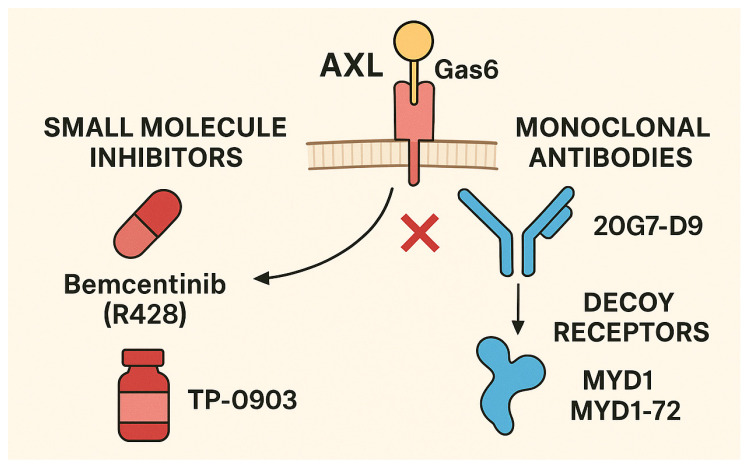
Axl inhibitors currently in development (suitable for the treatment of gastric cancer).

Monoclonal antibodies and decoy receptors represent another relevant class. Therapeutics targeting the extracellular Axl–Gas6 axis have advanced into preclinical and early clinical stages. 20G7-D9, an antibody against Axl, reduced Gas6-induced Akt activation, EMT marker expression, and tumor growth in pancreatic and breast cancer xenografts [52]. Additionally, antibody-drug conjugates (ADCs), such as Axl-107-MMAE and BA3011, combine Axl-targeting with cytotoxic payloads [53]. These ADCs demonstrated potent anti-tumor activity in vivo: Axl-107-MMAE was effective across cervical, lung, and melanoma models [54], and BA3011 impaired tumor growth in lung, ovarian, and prostate cancers [55]. Clinical trials of these ADCs are underway (e.g., NCT02988817, NCT03425279). Decoy receptors, such as MYD1 and MYD1-72, mimic the extracellular domain of Axl to sequester Gas6, effectively inhibiting ligand-mediated activation [56]. These agents have demonstrated tumor suppression and reduced metastasis in ovarian cancer preclinical models. AVB-500, another high-affinity Gas6 trap, is in phase I/II trials for ovarian and renal cell carcinoma [57]. Figure 3 offers an illustrative overview of Axl inhibitors currently in development.

### 4.2. Preclinical Evidence in Gastric Cancer

Preclinical studies have provided evidence supporting the therapeutic targeting of Axl in GC. A range of experimental approaches, including genetic silencing, small molecule inhibitors, and combination strategies, have demonstrated that Axl plays a crucial role in promoting tumor cell survival, proliferation, invasion, and resistance to therapy in both in vitro and in vivo models of GC.

One relevant preclinical study on Axl in GC was conducted by Bae et al. [58], who investigated the role of cancer-associated fibroblasts (CAF) in promoting aggressiveness in GC through the Axl signaling pathway. CAFs were found to produce high levels of GAS6, a ligand that activates the Axl receptor tyrosine kinase in GC cells. This activation increased GC cell migration, survival, and mesenchymal-like differentiation. Genetic inhibition of Axl or treatment with the Axl inhibitor BGB324 significantly reduced these effects. Additionally, high levels of phosphorylated Axl (P-Axl) in GC tissues correlated with poor patient survival. The findings suggest that targeting Axl with inhibitors like BGB324 could be a promising therapeutic strategy for GC [58].

The potential for pharmacological inhibition of Axl was further supported by studies using dual tyrosine kinase inhibitors targeting both MET and Axl. Given that co-activation of MET and Axl is commonly observed in gastric tumors, researchers evaluated the effects of combined inhibition in GC cell lines and xenograft models. A good example is the study by Zhu et al., which investigated the effects of LY2801653, a dual MET and AXL inhibitor, on gastric cancer. LY2801653 demonstrated inhibition of cancer cell proliferation, migration, and epithelial–mesenchymal transition (EMT), as well as induction of apoptosis and cell cycle arrest. In mouse models, it significantly reduced tumor growth, especially in tumors with high MET and AXL expression. At higher doses, LY2801653 also affected tumors lacking MET/AXL expression by reducing angiogenesis and M2 macrophages in the tumor microenvironment. Overall, the study supports MET and AXL as prognostic biomarkers and highlights LY2801653 as a promising therapeutic option for gastric cancer [56].

In addition to single-agent activity, Axl inhibitors have shown promise in combination with chemotherapeutic agents. Although much of the combination data stems from other malignancies, the mechanistic rationale applies strongly to gastric cancer. In models of breast and lung cancer, treatment with the selective Axl inhibitor R428 (bemcentinib) in combination with cisplatin resulted in enhanced tumor suppression and delayed metastatic spread compared to monotherapy. The synergy is thought to arise from the ability of Axl inhibition to prevent chemotherapy-induced activation of survival pathways such as AKT and STAT3, which are frequently upregulated in chemoresistant GC cells. Extrapolating to gastric cancer, where resistance to platinum and fluoropyrimidine-based regimens is a major clinical challenge, Axl inhibition may sensitize tumor cells to cytotoxic agents by disrupting the protective signaling networks [45].

Recent studies have also focused on identifying molecular subtypes of GC that may be particularly susceptible to Axl inhibition. Hu et al., 2024 [59] in their comprehensive (phospho) proteomic analysis of GC cell lines, used mass spectrometry to analyze protein and tyrosine phosphorylation patterns and performed clustering to identify GC subtypes. Among the key findings worth mentioning are the strong alignment between cell line proteomic profiles and patient-derived transcriptomic subtypes, the association of several protein kinases with abnormal expression or phosphorylation, including CSNK1D/E, with poor prognosis, the impact of the frequently hyperactivated Src family kinases, and GC cell lines showing broad sensitivity to the Src inhibitor eCF506. The two major GC subtypes are the EMT subtype, characterized by epithelial–mesenchymal transition and proliferation-related pathways, which are sensitive to mTOR inhibitors, and the metabolism subtype, characterized by enriched metabolic activity, cell junctions, and immune processes, and is sensitive to MAP2K2 (MEK2) inhibitors [59].

This preclinical data (summarized in Table 3) strongly supports the role of Axl as a therapeutic target in gastric cancer. Genetic and pharmacologic inhibition of Axl impairs multiple hallmarks of cancer and shows efficacy in both monotherapy and combination strategies. The enrichment of Axl activity in aggressive, mesenchymal GC subtypes further reinforces its value as a precision oncology target. Future preclinical studies should focus on validating the safety and efficacy of selective Axl inhibitors in advanced GC models, assessing their interaction with chemotherapy and immunotherapy, and identifying biomarkers—such as EMT status, Axl/Gas6 expression, or circulating soluble Axl—for patient selection and response monitoring. Given the encouraging in vivo results and mechanistic rationale, the transition from preclinical evidence to clinical trial evaluation of Axl-targeted therapies in gastric cancer appears both timely and warranted.

### 4.3. Clinical Trials

While most clinical development of Axl inhibitors has focused on other cancer types, the literature on GC remains scarce. Several trials have directly included gastric cancer (GC), particularly through dual or multi-kinase inhibitors with Axl activity. The most compelling GC-specific clinical data come from a phase I/II trial of LY2801653 (Merestinib), an oral ATP-competitive inhibitor targeting both MET and Axl. In a preclinical study by Zhu et al., LY2801653 demonstrated potent anti-proliferative, anti-EMT, anti-angiogenic, and apoptosis-inducing effects in gastric carcinoma models [58]. In MET- and Axl-high MKN45 xenografts, the agent significantly inhibited tumor growth. It also showed activity against MET/Axl-independent tumors at clinically relevant doses by modulating the tumor microenvironment and reducing M2 macrophage infiltration. This led to a documented phase I clinical trial (NCT01285037) in advanced solid tumors, which included gastric carcinoma patients, primarily to assess safety and establish dosing; however, efficacy data in GC are yet to be reported [60].

Another study evaluated CT053PTSA, a novel multi-target TKI against MET, Axl, and VEGFR2, in a phase I dose-escalation trial involving patients with advanced solid tumors, including GC. Preclinical work in SNU-5 and MKN-45 xenograft models demonstrated dose-dependent inhibition of tumor growth, along with excellent pharmacokinetic and safety profiles. The Phase I trial showed acceptable tolerability up to 150 mg daily, although recruitment was not restricted to patients with gastric cancer [61].

Additionally, cabozantinib, an oral TKI targeting MET, VEGFR2, and Axl, is being studied in combination with the anti-PD-L1 agent durvalumab in advanced gastroesophageal cancers, including gastric cancer, in a phase I/II open-label multi-cohort trial (NCT03539822). This study aims to evaluate the safety, tolerability, and preliminary efficacy, including response rates and progression-free survival, by leveraging both MET and Axl inhibition and immune modulation [36]. Furthermore, a clinical development program by BioSeedin is evaluating a selective c-Met/Axl inhibitor in a Phase I trial specifically enrolling gastric cancer patients; although published trial identifiers are not yet available, GlobalData confirms this candidate is in early-phase testing for GC.

In summary, while no trials to date have used Axl-selective agents in GC-exclusive cohorts, emerging data from multi-kinase inhibitors that include Axl strongly support its therapeutic relevance in gastric cancer. The safety and preliminary efficacy signals from Merestinib, CT053PTSA, cabozantinib, and the BioSeedin agent provide a solid foundation for future trials of selective Axl inhibitors or combination regimens in GC. Priorities for future clinical investigation should include selective Axl-targeted agents and combinations that exploit synergistic opportunities, such as immunotherapy or anti-MET approaches, in biomarker-enriched GC populations.

### 4.4. Combination Strategies

Recent preclinical studies have provided compelling mechanistic evidence that Axl inhibition enhances the efficacy of the PD-1/PD-L1 immune checkpoint blockade, offering a promising combination strategy for GC, where immune resistance and immunosuppressive microenvironments are current challenges.

One key study demonstrated that pharmacologic blockade or genetic knockout of Axl in murine syngeneic tumor models significantly potentiates antitumor immune responses when combined with anti–PD-1 therapy. In immunocompetent breast cancer models, Axl deletion resulted in increased CD8^+^ T-cell infiltration and tumor regression only when combined with PD-1 inhibition, leading to improved survival compared to either treatment alone. This outcome highlights Axl’s role in maintaining T-cell exclusion and immune evasion [62].

Similar results were reported by Yeo et al. [63] using the selective Axl inhibitor bemcentinib (BGB324) in an ovarian cancer model (ID8 cells). Axl blockade increased tumor-infiltrating effector CD4^+^ and CD8^+^ T cells, reprogrammed macrophages toward an M1 phenotype, and decreased immunosuppressive markers, such as Arginase-1 and IL-10. Although Axl inhibition alone had a modest effect, when combined with anti–PD–1, the dual treatment resulted in near-complete tumor rejection in several animals—an effect not observed with either single treatment.

At the molecular level, Axl signaling has been linked to upregulation of PD-L1 expression in multiple tumor types [64]. In lung and head-and-neck carcinoma cell lines, treatment with Axl inhibitors, such as bemcentinib or BGB324, downregulated PD-L1 and PD-L2 [65]. In contrast, in vivo Axl blockade activated antigen presentation pathways and increased CD8^+^ T-cell infiltration. This reversal of immunosuppression and reduction in checkpoint ligand expression may render tumors more susceptible to PD-1/PD-L1 blockade.

Moreover, studies in pancreatic and lung carcinoma models combining Axl inhibitors (e.g., SKI-G-801, BGB324) with standard chemotherapy and anti–PD–1 therapy have shown enhanced tumor control, associated with increased memory T cells and activated macrophages, alongside a reduction in regulatory T cells [66]. These results affirm that Axl-targeted approaches can restore chemo-immunogenicity and enhance immune-mediated killing.

Although direct clinical data in gastric cancer are pending, the pronounced immunomodulatory effects observed with Axl blockade in other tumor models—restoring T-cell presence, lowering checkpoint ligand expression, and improving response to PD-1/PD-L1 therapy—strongly support clinical exploration of this combination strategy in GC. Given the immunosuppressive tumor microenvironment and high PD-L1 expression in many GC tumors, integrating Axl inhibitors with immune checkpoint blockade holds significant promise. The incorporation of immune profiling, soluble Axl (sAxl) levels, and PD-L1 expression as biomarkers may further refine patient selection and enhance therapeutic outcomes in future clinical trials.

## 5. Axl, Tumor Microenvironment, and Therapeutic Implications

Another clinically and therapeutically relevant aspect of Axl is its influence, which extends beyond cancer cell-intrinsic signaling to significantly remodel the tumor microenvironment (TME). One of its primary ligands, GAS6, is abundantly secreted by tumor-associated macrophages (TAMs), endothelial cells, and fibroblasts within the TME [18]. The GAS6–Axl axis enhances tumor cell survival and invasion while fostering an immunosuppressive milieu. Axl activation in cancer cells induces the production of immunomodulatory cytokines and chemokines such as IL-10, TGF-β, and CCL2, which promote the recruitment and polarization of M2-like TAMs and regulatory T cells (Tregs), both of which contribute to immune suppression [27]. Moreover, Axl signaling in dendritic cells has been implicated in dampening antigen presentation and impairing T cell priming. In several types of cancer, high Axl expression is correlated with reduced cytotoxic T lymphocyte infiltration and increased expression of immune checkpoint molecules, such as PD-L1, thereby contributing to immune evasion [67]. This evidence highlights Axl as a critical mediator of immune resistance, positioning it as a rational target for combination therapy with immune checkpoint inhibitors (ICIs).

Axl functions as a central node in a complex signaling network and often engages in crosstalk with other receptor tyrosine kinases (RTKs), including MET, HER2, and EGFR [13]. These interactions confer signaling redundancy, promote oncogenic cooperation, and contribute to adaptive resistance mechanisms. In tumors, probably also in GC, co-activation of Axl and MET has been reported in aggressive subtypes, and dual inhibition has shown superior anti-tumor efficacy compared to monotherapies [68]. Axl forms heterodimers with MET, thereby amplifying downstream PI3K/AKT and MAPK signaling, which enhances cell proliferation, motility, and survival [69]. Similarly, Axl-HER2 interactions can sustain signaling even in the presence of HER2-targeted therapies, contributing to therapeutic resistance [13].

EGFR signaling is also modulated by Axl through both direct and indirect mechanisms. Axl can activate EGFR-independent pathways that converge on shared downstream effectors such as SRC, STAT3, and NF-κB, thereby sustaining oncogenic signaling under therapeutic pressure [70]. These compensatory mechanisms highlight the importance of targeting Axl-mediated signaling in combinatorial therapeutic strategies, particularly in tumors with co-dysregulation of RTKs.

Despite promising preclinical and early clinical data, resistance to Axl-targeted therapies has emerged as a significant challenge. Mechanisms of resistance include, in a great manner, secondary mutations. Mutations within the Axl kinase domain may reduce drug binding affinity or confer constitutive activation, resulting in the loss of inhibitor sensitivity [71]. Though not yet extensively characterized in GC, such mutations have been observed in other cancers and represent a potential barrier to long-term efficacy. Another mechanism involves bypassing signaling pathways. Redundant activation of parallel pathways, particularly through MET, EGFR, or FGFR, can circumvent Axl inhibition. These compensatory circuits reactivate PI3K/AKT and MAPK signaling, diminishing the impact of Axl-targeted agents. Upregulation of ligands such as Hepatocyte Growth Factor (HGF) or Epidermal Growth Factor (EGF) in the TME may further potentiate these bypass mechanisms [72].

The TME can directly support resistance through cytokine-mediated signaling or cellular interactions. For example, TAM-derived GAS6 can sustain Axl activation even in the presence of receptor blockades, while cancer-associated fibroblasts (CAFs) can modulate extracellular membrane (ECM) remodeling and stiffness, influencing receptor localization and signaling dynamics [73]. Additionally, immunosuppressive cell populations may persist, undermining anti-tumor immunity even when Axl signaling is inhibited. Overcoming resistance likely requires rational combination strategies targeting both tumor-intrinsic and TME-derived pathways, along with predictive biomarkers to identify patients most likely to benefit.

Axl expression and activation status could emerge as both prognostic and predictive biomarkers in GC. High Axl expression might be associated with advanced stage, lymph node metastasis, and reduced overall survival [74]. Phospho-Axl levels, reflecting active signaling, may serve as a more dynamic biomarker for therapeutic targeting. Still, clinical implementation requires rigorous statistical validation. Available studies are limited by small sample sizes and lack comprehensive reporting of effect estimates or *p*-values, which hampers definitive conclusions regarding their prognostic and predictive strength in GC. Future research should prioritize well-powered, statistically robust analyses that integrate hazard ratios, confidence intervals, and multivariate adjustments. Beyond Axl itself, biomarkers such as GAS6 expression, TAM density, and TME signatures (e.g., M2 macrophage markers, Treg infiltration) may provide insight into the broader Axl signaling axis [14]. Integrative molecular profiling, including proteomics, transcriptomics, and phosphoproteomics, can identify Axl-dependent subtypes and co-altered pathways, enhancing precision in patient stratification.

Emerging data also suggest that co-expression of Axl and other RTKs (e.g., MET, HER2) may predict sensitivity to dual or multi-kinase inhibition strategies [74]. Furthermore, gene expression signatures linked to epithelial–mesenchymal transition (EMT)—a phenotype commonly driven by Axl—may serve as indirect indicators of Axl pathway activation and therapy responsiveness.

## 6. Conclusions and Future Directions

With the increasing unfavorable epidemiological data related to GC, novel biomarkers and therapeutic targets must be explored. In this context, Axl could be a desirable molecule that plays a significant role in the pathogenesis and progression of gastric cancer by promoting tumor cell survival, proliferation, epithelial-to-mesenchymal transition, angiogenesis, and therapeutic resistance. Its activation through ligands such as Gas6 triggers multiple oncogenic pathways, contributing to immune evasion and tumor aggressiveness. Clinically, Axl overexpression might correlate with advanced disease features and poor prognosis, making it a valuable prognostic biomarker and potential therapeutic target. The soluble form of Axl (sAxl), detectable in blood, also could show promise as a non-invasive biomarker for disease monitoring. Together, these insights underscore the importance of Axl in gastric cancer biology and support ongoing efforts to target this pathway in precision oncology strategies.

Therapeutic targeting of Axl in GC has gained momentum, supported by preclinical data and emerging clinical evidence. Small-molecule inhibitors, monoclonal antibodies, and decoy receptors have demonstrated efficacy in inhibiting Axl-driven processes, including EMT, metastasis, and immune evasion. Dual MET/Axl inhibitors, such as LY2801653 and CT053PTSA, have shown promising activity in gastric cancer models and early-phase trials. Furthermore, combination strategies—particularly those involving immune checkpoint inhibitors—have demonstrated synergistic effects in various tumor types, thereby enhancing CD8^+^ T-cell infiltration and reversing immunosuppression. These findings underscore the potential of Axl inhibition, both as monotherapy and in rational combinations, to overcome resistance and improve clinical outcomes in gastric cancer. Still, future trials should focus on biomarker-guided patient selection, including Axl expression and EMT status, to optimize therapeutic benefit, as a discrepancy currently exists between preclinical and clinical results.

Furthermore, Axl plays a critical role in reshaping the tumor microenvironment in gastric cancer, promoting immune evasion, therapy resistance, and tumor progression through the GAS6–Axl axis. By fostering an immunosuppressive milieu—characterized by increased M2-like macrophages, Tregs, and reduced antigen presentation—Axl contributes to poor immune infiltration and elevated PD-L1 expression, making it a prime candidate for combination with immune checkpoint inhibitors. Its extensive crosstalk with MET, HER2, and EGFR underscores its role in signaling redundancy and resistance to targeted therapies. However, resistance to Axl inhibitors can arise via secondary mutations, bypass signaling, and persistent TME-mediated activation. As both a prognostic and predictive biomarker, Axl expression, along with phospho-Axl, GAS6 levels, and EMT-related gene signatures, can inform patient stratification and therapeutic response. These insights advocate for integrative, biomarker-driven approaches to Axl-targeted therapy, especially in combination with RTK or immune-based regimens, to overcome resistance and optimize outcomes in gastric cancer.

The development of more selective and potent Axl inhibitors represents a critical next step in advancing targeted therapies for gastric cancer. Current agents, while promising, are often limited by off-target effects, resistance mechanisms, and suboptimal activity in biomarker-unselected populations. To accelerate clinical translation, several research priorities should be emphasized. First, comprehensive biomarker development is essential, including prospective validation of Axl expression, phospho-Axl activity, and circulating sAxl levels as predictive tools for patient selection. Second, rational trial design should integrate Axl inhibitors with standard-of-care chemotherapy, immunotherapy, or HER2/MET inhibitors, guided by preclinical evidence of synergy. Furthermore, mechanistic studies are needed to clarify resistance mechanisms, such as compensatory RTK activation or TME-mediated immunosuppression, that may limit therapeutic efficacy. Finally, early-phase, biomarker-enriched gastric cancer trials should be prioritized to bridge the gap between promising preclinical findings and clinical applicability. Additionally, comparative multi-omics approaches (proteomics, phosphoproteomics, and single-cell transcriptomics) should be applied to identify GC-specific Axl-driven subtypes, which may facilitate the development of precision strategies rather than broad, unstratified treatment approaches. Future efforts should prioritize the design of next-generation inhibitors with enhanced specificity, improved pharmacokinetics, and the ability to overcome known resistance pathways. Additionally, alternative strategies such as antibody–drug conjugates, bispecific antibodies, and ligand traps offer complementary avenues to inhibit Axl signaling. Incorporating these innovations into biomarker-guided clinical trials will be essential to fully realize the therapeutic potential of Axl targeting in gastric cancer.

## Figures and Tables

**Table 1:** Most relevant roles of Axl in (gastric) cancer pathophysiology—a qualitative analysis.

Biological Process	Mechanism of Axl Involvement	Related Pathways	Potential Implications in GC
Cell proliferation and survival	Activation of PI3K/AKT and MAPK/ERK pathways via recruitment of p85 and Grb2	PI3K, AKT, MAPK, ERK	Promotes cell cycle progression, inhibits apoptosis, supports tumor growth
Apoptosis resistance	Upregulates anti-apoptotic proteins (e.g., BCL-2, Cyclin D1), downregulates pro-apoptotic proteins (e.g., BAX)	AKT, STAT3	Confers resistance to cisplatin and other chemotherapies
Migration and invasion (EMT)	Induction of EMT transcription factors (Snail, Twist); modulation of cell adhesion and cytoskeletal dynamics	Vimentin, N-cadherin, E-cadherin, Rac1, RhoA, FAK	Enhances metastatic potential and invasiveness
Angiogenesis	Induces pro-angiogenic cytokines (e.g., VEGF, IL-8); enhances crosstalk with endothelial cells	VEGF, IL-8, pericyte interactions	Supports tumor vascularization and growth
Therapeutic resistance	Upregulation after treatment; heterodimerization with HER2, MET, FGFR2 sustains survival signaling despite therapy	PI3K/AKT, MAPK, HER2, MET, FGFR2	Resistance to trastuzumab, TKIs, and chemotherapy; associated with poor outcomes

**Table 3:** Preclinical evidence for Axl targeting in (gastric) cancer (→—determines the following process; ↑—increase in value).

Study	Design/Approach	Most Relevant Conclusions	Potential Therapeutic/Translational Implications
Bae et al., 2020 [57]	GC cell lines + CAF co-culture; Axl inhibition (genetic, BGB324); patient tissue analysis	CAF-derived GAS6 activates Axl → ↑ migration, survival, EMT; Axl inhibition reverses effects; P-Axl correlates with poor prognosis	Supports Axl inhibition (e.g., BGB324) as a strategy to target CAF-driven aggressiveness in GC
Zhu et al., LY2801653 study [58]	Dual Axl/MET inhibitor in GC cell lines and xenografts	Inhibits proliferation, migration, EMT; induces apoptosis; decreased tumor growth in high MET/AXL models; decreased angiogenesis and M2 TAMs	Dual MET/AXL inhibition may benefit Axl+/MET+ GC; reduces tumor microenvironment support
R428 + chemotherapy (cross-cancer data) [45]	Breast/lung cancer models; extrapolated to GC	Axl inhibition prevents AKT/STAT3 activation by chemotherapy; enhances cisplatin efficacy and delays metastasis	Supports combining Axl inhibitors with standard chemotherapy in GC to overcome drug resistance
Hu et al., 2024 (Phosphoproteomics) [59]	MS-based analysis of GC cell lines and subtypes	Identifies EMT and metabolism subtypes; Axl activity enriched in EMT subtype; subtype-specific kinase vulnerabilities	Axl inhibitors may be especially effective in mesenchymal/EMT-enriched GC subtypes

## Data Availability

All data are available in the manuscript.
